# A Rare Complication of a TAP Block Performed after Caesarean Delivery

**DOI:** 10.1155/2017/1072576

**Published:** 2017-10-29

**Authors:** Osman Nawazish Salaria, Murlikrishna Kannan, Bryan Kerner, Howard Goldman

**Affiliations:** Department of Anesthesiology, Mount Sinai Medical Center of Florida, Miami Beach, FL 33139, USA

## Abstract

The transversus abdominis plane block is a regional anesthesia technique that has become popular. Being a relatively simple procedure, the TAP block has an excellent safety profile and major complications are rare. We present a case of transient femoral nerve palsy occurring after a TAP block with involvement of the sacral plexus for a patient who had undergone a caesarean section.

## 1. Introduction

The transversus abdominis plane block is a regional anesthesia technique that has recently become more popular for two reasons: the use of ultrasound to improve block placement and the desire to reduce the need for perioperative narcotics. It has been used in surgeries involving the anterior abdomen (e.g., colorectal surgery, caesarean section) as an effective and reliable technique to improve postoperative analgesia [1–3]. The targets of this procedure are the sensory nerves originating from T7 to T11 that innervate the abdominal wall (intercostal, ilioinguinal, subcostal, and iliohypogastric nerves) [2–5]. Being a relatively simple procedure, the TAP block has an excellent safety profile and major complications are rare. There have been reports of local anesthetic systemic toxicity, bowel hematoma, and liver laceration [3, 6]. Transient femoral nerve palsy is another unusual complication of this procedure. The mechanism behind the development of this complication is unclear, but theories have been proposed [2, 3]. We present a case of transient femoral nerve palsy occurring after a TAP block for a patient who had undergone a caesarean section.

## 2. Case Report

A 29-year-old female, Gravida 1, Para 0, at 39 + 1 weeks of gestational age, presented for a primary caesarean section. The patient had a medical history of hip disease and her orthopedic physician recommended she have a caesarean section to avoid hyperflexion of the hip. A firm allergy to narcotic pain medications was noted; thus they were avoided. The patient was counseled on the TAP procedure for postoperative pain control as an alternative, which she elected for. Medication history included prenatal vitamins along with Prilosec for occasional acid reflux. A spinal neuraxial block was performed for anesthesia. 7.5% povidone-iodine solution was used for sterilization. After the patient was draped and prepped, a 25 G × 10 cm Sprotte needle was used for identification of the intrathecal space. 1.3 cc of 0.75% bupivacaine along with 15 mcg of fentanyl and 0.3 mg of morphine was injected after free flow of CSF was obtained. The patient's sensory level after neuraxial block was identified to be at the T5 level bilaterally. The caesarean section was performed by the obstetrician and a healthy neonate was delivered. APGAR scores of 9/9 were recorded at 0 and 5 minutes, respectively. A TAP block was then performed for postoperative pain control after the obstetrician had sutured the uterine incision. Chlorhexidine was used for sterile preparation and the patient was placed in the supine position. The ultrasound probe was placed between the costal margin and the iliac crest. All 3 layers of the abdominal wall were visualized. A 20 G × 4 in Braun needle was used for piercing the superficial structures and the oblique muscles. The needle was inserted in plane with the ultrasound probe and directed towards the fascia between the internal oblique and transversus abdominis muscles. An amount of 25 cc of 0.5% bupivacaine with 1 : 200,000 epinephrine was injected bilaterally between the internal oblique and transversus abdominis muscles as a single shot technique. An abdominal binder was placed for support and the patient was taken to the PACU. Initial vital signs on arrival were blood pressure of 104/77, heart rate of 65, temperature of 98.4, and respiratory rate of 15 with 100% saturation. After a time period of 1 hour in the PACU, the patient was reassessed and noted to be stable. Vital signs were BP of 132/80, HR of 91, temperature of 98.3, and RR of 19 with 100% saturation. The patient had adequate analgesia and was able to move her distal extremities. Using the Modified Aldrete Criteria, the patient was discharged from the PACU. On postoperative day 1, the anesthesia team followed up with the patient. She complained of new onset paresthesias in the bilateral medial thighs as well as left lower extremity motor weakness to be present. She was not able to ambulate and complained of mild pain well controlled with Toradol. Physical examination denoted left lower extremity to have 0/5 strength left thigh flexion, 2/5 strength leg extension, and 2 + patellar reflexes. Dorsiflexion and plantar flexion movements were noted to be reduced in strength for the left lower extremity. Right lower extremity exam showed that she had 3/5 thigh flexion, 4/5 strength leg extension, and 2 + patellar reflexes. Dorsiflexion and plantar flexion was noted to be intact for the right lower extremity. Sensation to crude touch and pin prick sensation were intact bilaterally in both extremities. Sensory examination showed intact sensation to crude touch and pin prick sensation in bilateral lower extremities. The patient was reassured and told that symptoms were temporary. Further follow-up on postoperative day 2 showed that the patient had full recovery of sensation and strength in bilateral lower extremities. The patient was ambulating well and a numeric pain scale of 3/10 was observed.

We postulate that the patient developed transient bilateral femoral nerve palsy with involvement of the sacral plexus on postoperative day one. A combination of a large volume of local anesthetic and the pressure on the abdomen by the abdominal binder likely contributed to this complication.

## 3. Discussion

The transversus abdominis plane block is a regional anesthesia technique that has been used as part of a multimodal approach to control postoperative pain. Procedures involving the anterior abdominal wall such as colorectal, laparoscopic, and gynecological operations have shown benefits with this procedure [1, 3, 7, 8]. Several randomized controlled trials have shown the reduction in postoperative opioid consumption, improved patient satisfaction, and early ambulation [3, 7]. The incidence of pruritis and postoperative pain scores were reported as much lower [7]. These studies did not show improvements in postoperative sedation and postoperative nausea and vomiting [1]. However, when compared to control groups, patients who received the TAP block had an overall higher level of satisfaction [1]. Complications from this procedure have been few. Intravascular injection, local anesthetic systemic toxicity, and lacerations from enlarged liver/spleen have been noted [2, 3, 6, 9]. With ultrasound guidance, these complications have been reduced. Transient femoral nerve palsy has also occurred and is presented in this case report. The anatomy of the abdominal wall and continuity of fascial planes has shown that this complication can occur more frequently. The fascia transversalis continues with the fascia iliaca in the abdominal wall posterolaterally [2, 3]. If incorrect injection of local anesthetic is made between the transversus abdominis muscle and fascia transversalis, it can track down the fascia and accumulate around the femoral nerve. This will lead to weakness in the lower extremities as well as affecting ambulation of the patient, as in our case.

Although correlation with the type and volume of local anesthetic with the incidence of femoral nerve palsy has not been shown, we suspect that longer acting agents might increase the incidence. In our study, a large volume of bupivacaine with epinephrine was used, 0.5%, injecting 25 cc's bilaterally. A similar study performing a TAP block for inguinal herniorrhaphy showed sensory blockage with usage of 20 cc's of 0.5% bupivacaine with epinephrine, without motor involvement [3]. With the presence of the abdominal binder, we hypothesize that the pressure on the abdomen caused the spread of local anesthetic to travel down the fascial planes and accumulate around the femoral nerve. This was seen on postoperative day one with bilateral lower extremity weakness and inability to ambulate. The effects were transient and the patient had gained full recovery of sensory and motor function of both lower extremities by postoperative day two.

## 4. Conclusion

The TAP block is a safe and effective procedure for postoperative pain relief. The complications of this procedure are low and preventable. This case report illustrated a rare complication of transient femoral nerve palsy. A combination of high volume and concentration of local anesthetic and the pressure of the abdominal binder resulted in the development of this complication.
